# Role of Non-conventional T Lymphocytes in Respiratory Infections: The Case of the Pneumococcus

**DOI:** 10.1371/journal.ppat.1004300

**Published:** 2014-10-09

**Authors:** Stoyan Ivanov, Christophe Paget, François Trottein

**Affiliations:** 1 Institut Pasteur de Lille, Centre d'Infection et d'Immunité de Lille, Lille, France; 2 Institut National de la Santé et de la Recherche Médicale, U1019, Lille, France; 3 Centre National de la Recherche Scientifique, UMR 8204, Lille, France; 4 Université Lille Nord de France, Lille, France; University of Florida, United States of America

## Abstract

Non-conventional T lymphocytes constitute a special arm of the immune system and act as sentinels against pathogens at mucosal surfaces. These non-conventional T cells (including mucosal-associated invariant T [MAIT] cells, gamma delta [γδ] T cells, and natural killer T [NKT] cells) display several innate cell-like features and are rapidly activated by the recognition of conserved, stress-induced, self, and microbial ligands. Here, we review the role of non-conventional T cells during respiratory infections, with a particular focus on the encapsulated extracellular pathogen *Streptococcus pneumoniae*, the leading cause of bacterial pneumonia worldwide. We consider whether MAIT cells, γδ T cells, and NKT cells might offer opportunities for preventing and/or treating human pneumococcus infections.

## Introduction

*Streptococcus pneumoniae* (commonly referred to as “pneumococcus”) is an important human pathogen that causes severe pulmonary and invasive diseases. Over the past decades, progress has been made in (i) the understanding of host innate and acquired immunity during *S. pneumoniae* infection and (ii) the development of antipneumococcal vaccines. Non-conventional T lymphocytes (including mucosal-associated invariant T [MAIT] cells, gamma delta [γδ] T cells, and natural killer T [NKT] cells) are antigen (Ag)-reactive immune cells with important innate-like functions. In the present review article, we discuss recent advances in our understanding of the innate-like mechanisms underlying the activation of nonconventional T cells and consider their putative roles in pneumococcal infection and disease.

## Pneumococcus: A Major Respiratory Pathogen Worldwide

Pneumococcal infection causes around two million deaths per year and is associated with a huge economic burden. Community-acquired pneumonia caused by pneumococci accounts for more than 25% of all cases of pneumonia (for reviews, [1]–[3]). *S. pneumoniae* is an encapsulated, gram-positive, extracellular bacterium. More than 90 serologically and biochemically distinct serotypes (based on the structure of the bacterial capsule) have been described; they differ in terms of invasiveness, virulence, and antibiotic resistance [1]–[4]. In healthy individuals, *S. pneumoniae* colonizes the upper respiratory tract but does not appear to have an obvious negative impact. However, in people with an immature or compromised immune system, this asymptomatic colonization can progress to mild disease (such as sinusitis and otitis media) and occasionally to pneumonia, sepsis, and meningitis [3]–[5]. The incidence of pneumococcal infections depends on a number of parameters, including bacterial virulence factors (i.e., the nature of the polysaccharide capsule and the presence or absence of the exotoxin pneumolysin) and host factors (i.e., smoking habit, immune status, and history of respiratory infections) [6]–[9]. For example, influenza infection leads to enhanced susceptibility to pneumococcal infection, a major cause of deaths during influenza epidemics and pandemics [10], [11]. The relative inefficacy of antibiotics is a major issue in pneumococcal infection post-influenza. Furthermore, an increasing number of antibiotic-resistant strains are now emerging [12], [13]. Enhanced susceptibility to pneumococcal infection also occurs during conditions with chronic lung inflammation such as chronic obstructive pulmonary disease [14], which is forecast to become the third most common cause of death worldwide by 2020 [15].

Vaccination is an efficient strategy for preventing and controlling pneumococcal infections, although currently available vaccines do have some issues (for reviews, [16]–[18]). For instance, the 23-valent vaccine that contains purified capsular polysaccharides does not adequately protect young children under two years old or the elderly ([18], [19]). The main reason is that capsular polysaccharides are T-cell independent Ags and are therefore poorly immunogenic. The 7-valent vaccine (containing polysaccharides conjugated to protein carriers, to enhance immunogenicity) is associated with a reduction in the number of invasive pneumococcal diseases, but only those involving the seven serotypes included in the formulation [20]. At present, a 13-valent vaccine is used for infants. In the future, alternative pneumococcal vaccines (directed against virulence factors shared by numerous serotypes and coupled to adequate adjuvants) are likely to be developed [17].

## Nonconventional T Lymphocytes

Over the last few years, interest in understanding the role of nonconventional T lymphocytes in immune homeostasis and disease has grown tremendously. These “innate-like” T cells differ from conventional, adaptive T lymphocytes in many respects (Table 1). When nonconventional T lymphocytes emerge from the thymus, they are already capable of cytolysis and cytokine release. The ability to exert effector function soon after activation suggests that nonconventional T cells occupy a unique niche in the immune system (between innate and adaptive immunity). In contrast to the huge receptor diversity of conventional T cells, the T cell receptor (TCR) expressed on the surface of nonconventional T cells presents a limited number of rearrangements and only recognizes conserved, nonpeptide Ags. On the basis of this definition, nonconventional T lymphocytes correspond to three major cell types: MAIT cells, γδ T cells, and NKT cells (Table 1).

**Table 1:** Differences between conventional T cells and non-conventional T cells.

Feature		TCR repertoire	Restriction reactivity	TCR ligands	Markers and subsets	Frequency and location	Response kinetics
**Conventional T cells**		Highly variable Diverse αβ TCRs	MHC class I and II Highly polymorphic	Processed peptides	CD4^+^ (MHC II) CD8^+^ (MHC I) CD4^−^CD8^−^	Blood Lymphoid tissues	Late (after clonal expansion) Cytokines Cytotoxic activity (CD8^+^)
**MAIT cells**		Semi-invariant Invariant α-chain Vα7.2-Jα33 (humans) Vα19-Jα33 (mice) Restricted number of β chains [25], [26]	MR1 [21]	Unprocessed Vitamin B_2_ metabolites (pterin analogues) [23], [27]	Two major subsets (pathogen reactive versus immune modulatory functions [31], [32]	Mucosal sites Gut and lung Liver Blood (1–10% of PBMC) [21]–[24]	Immediate Cytokines Cytotoxic activity [23], [28]–[30], [36], [37]
**γδ T cells**	*Mouse*	Restricted including some clonal TCRs (Vγ1Vδ6.3, Vγ5Vδ1, Vγ6Vδ1) [38]	CD1d, T10/T22, butyrophilin 3A1 [38]–[40], [42]	Wide range Cardiolipin, Phycoerythrin, Insulin peptide [51], [52]	NKR, CD27, NK1.1, CCR6 [38]	Mucosal sites Epidermis [38]	Early Cytokines High cytotoxic activity [38], [54], [65], [70]
	*Human*	Semi-invariant or variant Restricted number of γ and δ chains [38], [40]	MHC-related (CD1d, CD1c and MICA/B) MHC-unrelated (including viral glycoproteins, F1-ATPase complex) [38], [48], [49]	Unprocessed PhosphoAgs, Phycoerythrin, Glycolipids (sulfatide and α-GalCer (Vδ1^+^ cells)), EPCR and others [46]–[49], [51], [53]	NKR CD4^+^ CD8^+^ (70% DN, 30% CD8^+^αα (as IELs in gut) [38]	Mucosal sites Blood (2–10% of T cells, mainly Vγ9Vδ2) [38]	Early Cytokines High cytotoxic activity [38], [50], [61], [62]
**NKT cells**		Semi-Invariant Invariant α-chain Vα24-Jα18 (humans) Vα14-Jα18 (mice) Restricted number of β chains [77]–[79]	CD1d [78]	Unprocessed or processed Glycolipids Phospholipids [80], [82]	NKR CD4^+^/CD8 Few subsets based on CD4, CD8 (human), NK1.1 and IL-25R [90]	Liver Mucosal sites Blood (0.1–0.01% of PBMC) [78], [79], [84]	Early Cytokines Cytotoxic capacity [78]

Abbreviations: PMBC, peripheral blood mononuclear cell; NKR, Natural Killer cell Receptor; EPCR, Endothelial protein C Receptor.

### MAIT cells

#### General characteristics

In humans, MAIT cells account for between 1% and 10% of T cells in the peripheral circulation [21]. They are also found in liver and mucosal tissues, including the intestine (lamina propria) and the lung [22]–[24]. Although murine MAIT cells also populate lymphoid and mucosal tissues, their frequency is much lower than in humans [25]. MAIT cells express a semi-invariant TCR, which comprises an essentially invariant TCR α-chain (Vα7.2-Jα33 in humans and Vα19-Jα33 in mice) that is preferentially paired with Vβ2, Vβ13, or Vβ22 segments in humans and Vβ6 or Vβ8 segments in mice [25], [26]. The MAIT TCR is restricted to Ags presented by the monomorphic major histocompatibility complex (MHC) class I-related molecule MR1, which is highly conserved in mammals [21]. Recent research has demonstrated that metabolites of riboflavin (also known as vitamin B_2_) are MR1-dependent ligands for MAIT cells [27]. Unlike certain microorganisms, mammals do not possess the enzymatic machinery needed to generate these riboflavin compounds. Hence, MAIT-cell–activating riboflavin Ags are generated by pathogenic and commensal bacteria [23], [28], the latter being essential for the development and expansion of MAIT cells [21], [22], [28]. In view of their physiological sites, this observation suggests that MAIT cells are important in the early detection of microbial infection and the subsequent elicitation of a successful immune response. Along with TCR-mediated stimulation, cytokines (IL-12, IL-1β, IL-23) are instrumental in amplifying MAIT cell activation [29], [30]. MAIT cells are able to swiftly exert their effector functions upon activation and therefore make an important contribution to the immune response. The role of MAIT cells in health and disease is becoming clearer. A growing body of evidence indicates that MAIT cells are involved in autoimmune disorders, rheumatoid arthritis, intestinal inflammation, and infection (for reviews, [31]–[34]).

#### Role during respiratory infections

Human and/or mouse MAIT cells are activated when host cells become infected by certain bacteria (i.e., *Salmonella enterica*, *Pseudomonas aeruginosa*, *Staphylococcus aureus*, *Escherichia coli*, *Klebsiella pneumoniae*, *Lactobacillus acidophilus*, *Shigella flexneri*, *Mycobacteria*) and yeasts (*Candida* and *Saccharomyces*) but not by viruses [23], [28], [35]. This activation is MR1-dependent and leads to the production of TNF-α and IFN-γ, cytokines known to control intracellular infection. In contrast, *Enterococcus*, *Listeria monocytogenes*, and group A *Streptococcus* (*S. pyogenes*) fail to activate MAIT cells [23], [28], suggesting a defect in the enzymatic pathway leading to the generation of riboflavin metabolites in these bacterial species. Several clinical studies have investigated the dynamics and activation status of MAIT cells in patients with pulmonary bacterial pathologies (especially in the context of tuberculosis) [23], [28]. Patients with tuberculosis displayed a significant decrease in the frequency and number of circulating MAIT cells [28]. Moreover, MAIT cells were virtually absent in the blood of patients with active tuberculosis [23]. This is probably due to their recruitment and, possibly, subsequent expansion in the lung tissue and pleural effusions of patients [23], [28]. MAIT cells recognize and kill *Mycobacterium tuberculosis*-infected cells, including dendritic cells (DCs) and lung epithelial cells [23], [36]. Murine MAIT cells are also important in innate immunity against mycobacteria [29]. Through IFN-γ secretion, they can efficiently inhibit the growth of *Mycobacteria bovis* bacillus Calmette-Guerin (BCG) in macrophages [29]. In this setting, MAIT cell activation relies on MR1 recognition and co-signals, including IL-12 released from infected macrophages. *M. bovis* BCG–infected *Mr1* ^−/−^ mice displayed a higher bacterial burden in the lung than MAIT cell–proficient animals [29]. *Francisella tularensis* is a highly virulent, ulcerative, gram-negative bacterium that causes tularaemia. During infection, murine MAIT cells expand gradually in the lungs; this is accompanied by the secretion of IL-17, IFN-γ, and TNF-α [37]. As observed in the murine *M. bovis* BCG model, MAIT cell deficiency leads to a delay in the control of bacterial growth. The role of MAIT cells in the development of the acquired immune response has yet to be defined. It is noteworthy that after *F. tularensis* challenge, mice lacking MAIT cells display fewer activated pulmonary CD4^+^ and CD8^+^ T cells. Although the specificity of these conventional T cells was not formally addressed, this finding suggests that MAIT cells have a role in the adaptive immune response [37].

### γδ T cells

#### General characteristics

Several subtypes of γδ T cells have been described. They differ according to the rearrangement of the TCR during early development in the thymus (for reviews, [38]–[40]). Subsets of γδ T cells bearing specific γ and δ TCR chains are assigned to particular body sites. For example, the majority of human γδ T cells in the blood (2%–10% of peripheral T cells) carry a Vγ9Vδ2 TCR, whereas the expression of other Vδ elements (particularly Vδ1 and Vδ3 TCR) predominates at epithelial surfaces and mucosae. This infers homogeneous but distinct Ag recognition repertoires and functional specificities when comparing one tissue with another. In the mouse system, γδ T cells with distinct Vγ/Vδ usage are present in lymphoid tissues (Vγ1^+^ and Vγ4^+^ cells), skin (Vγ5^+^ cells), and mucosal tissues such as the intestine (Vγ7^+^ cells), reproductive tract (Vγ6^+^ cells), and lung (Vγ1^+^, Vγ4^+^ and Vγ6^+^). In the lung, γδ T cells are uniformly distributed in parenchymal and non-parenchymal sites [41].

**Figure 1:**
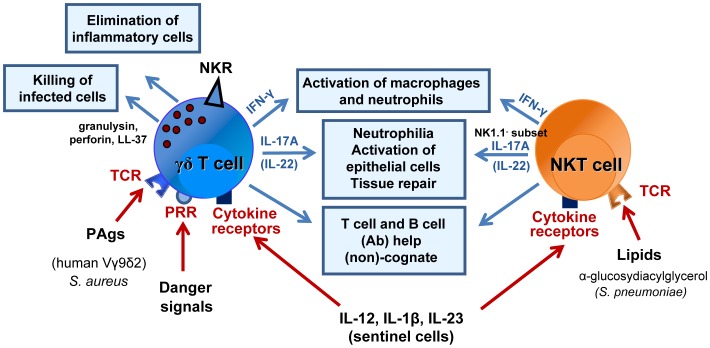
Mode of activation and role of γδ T cells and NKT cells during bacterial respiratory infections. γδ T cells and NKT cells are activated through the TCR, cytokine receptors and/or PRRs (at least for γδ T cells). Their protective role during respiratory bacterial infections (*S. aureus*, *M. tuberculosis*, *C. pneumoniae*, *S. pneumoniae*) is diverse and comprises activation of innate effector cells, such as macrophages and neutrophils (IFN-γ, IL-17), and epithelial cells (IL-17, IL-22) or direct killing of infected cells (γδ T cells). At later time points, γδ T cells and NKT cells might also play a crucial role in tissue repair, for instance, by acting on epithelial cells and/or by eliminating inflammatory cells. They also promote the development of acquired immune responses. Activation and expansion of T lymphocytes and B lymphocytes (Abs) can occur in a non-cognate way through cytokine release and activation of dendritic cells or in a cognate manner (see Figure 3). Abbreviations: NKR, NK cell receptor; TCR, T cell receptor; PRR, pattern recognition receptor.

Gamma delta T cells are uniquely equipped to sense cellular stress-induced ligands via both TCR-dependent and -independent pathways (Figure 1). These danger-associated molecular pattern-like structures can be expressed during infection, transformation and inflammation. Activation of γδ T cells via TCRs can be mediated by non-classical MHC molecules (i.e., T10/T22 and CD1 family members) and MHC-unrelated molecules (i.e., viral glycoproteins and butyrophilin 3A1) [38]–[40], [42]. The nature of Ags recognized by γδ TCRs is diverse and still incomplete. Human Vγ9Vδ2 T cells react to phosphoAgs derived from the mevalonate pathway [43]–[46]. Human γδ (Vδ1^+^) T cells recognize lipids presented by CD1 molecules [47]–[49], as well as unprocessed proteins including viral proteins, phycoerythrin, insulin peptide, and stress-induced molecules [50]–[53]. In addition to the TCR, γδ T cells express a wide array of nonclonal receptors, including pattern recognition receptors (PRRs) and natural killer (NK) cell receptors. Regarding the latter group, γδ T cells can sense stressed-induced ligands (Rae1 and MICA/B) through NKG2D engagement. Gamma delta T cells are also directly activated through Toll-like receptors (TLRs) and C-type lectins (including dectin-1) [54] and indirectly activated by microbial products via Ag-presenting cells. In this context, the release of pro-inflammatory cytokines by stressed DCs activates γδ T cells in the absence of additional TCR engagement [54]–[58]. These different modes of activation suggest that γδ T cells are involved in many disease situations. Lastly, γδ T cells have a crucial role in immune surveillance against tumours, protective immunity against pathogens, inflammation, tissue healing, and epithelial cell maintenance (for reviews, [38]–[40], [59], [60]). This multifaceted role of γδ T cells is due to (i) the existence of distinct γδ subsets endowed with specific functions and (ii) functional polarization in the periphery under stressful conditions. Gamma delta T cells produce a wide array of cytokines and display potent cytotoxic activities against infected or transformed cells, with effector functions via apoptosis-inducing receptors (FAS and TRAIL) and cytolytic proteins (perforin and granzyme) [61], [62]. In addition to their beneficial mediation of local immune surveillance, γδ T cells can also contribute to the immunopathology and progression of diseases, including autoimmune diseases [63].

#### Roles during respiratory infections

Systemic infections can lead to γδ T cell activation and expansion. Gamma delta T cells mediate pathogen clearance directly through the production of bacteriostatic and lytic molecules, such as granulysin and defensins [61], [64], and indirectly through cytokine-mediated activation of effector cells including macrophages and neutrophils [65]. Pulmonary γδ T cells are uniformly distributed in the lungs, where they are closely associated with Ag-presenting cells [41]. Gamma delta T cells have a role during respiratory infections. For example, the activation of human Vγ9Vδ2 T cells by endogenous mevalonate metabolites during influenza A virus (IAV) infection leads to virus clearance through NKG2D engagement and IFN-γ secretion [62], [66]–[68]. Gamma delta T cells also contribute to the clearance of intracellular and extracellular respiratory bacteria (Figure 1). During *S. aureus* infection, Vγ9Vδ2 T cells sense the dysregulation of the mevalonate pathway within infected cells—a mechanism that might lead to staphylococcal immunity [69]. In mice, *S. aureus* triggers IL-17 production by γδ T cells, thus leading to protection and airway inflammation [70]. Gamma delta T cells become activated and expand during *M. tuberculosis* infections in mice [71], [72]. Whereas IFN-γ and TNF-α derived from γδ T cells might be protective against *M. tuberculosis* infection, IL-17 production by Vγ4^+^ and Vγ6^+^ γδ T cells might induce granuloma formation [73]–[76]. In humans, peripheral blood γδ T cells are a major source of IL-17 during active pulmonary tuberculosis; however, the functional significance of this secretion is not clear [72].

### NKT cells

#### General characteristics

Natural killer T cells recognize a broad range of endogenous and exogenous lipid Ags. These Ags are presented by the monomorphic CD1d molecule expressed by Ag-presenting cells such as DCs [77], [78]. CD1d-restricted NKT cells are divided into two major subsets on the basis of their TCR repertoire and antigenic profile (for reviews, [78]–[81]). The best-characterized NKT cell subset by far is the “type I NKT cell” population. These cells express a restricted TCR, within which an invariant α chain (Vα24-Jα18 in humans and Vα14-Jα18 in mice) is combined with a limited Vβ-chain repertoire (Vβ8.2, Vβ7, or Vβ2 in mice and Vβ11 in humans) [77]. An important feature of type I NKT cells is the rapid release of cytokines and chemokines. This phenomenon is responsible for early effector and regulatory functions. The second major subset of NKT cells (“type II”) expresses a more diverse TCR repertoire and recognize different CD1d-associated lipids [80], [82]. This population may be important in auto-immunity, infections, and cancer [83], although the current lack of specific molecular markers is hindering research in this field. In the present review, we shall focus on type I NKT cells (referred to henceforth as “NKT cells”).

Natural killer T cells are present at higher frequencies than MAIT cells and γδ T cells in mice but at lower frequencies in humans. Human NKT cells comprise a small proportion of peripheral T cells (∼0.1%–0.01% in humans) and vary in their frequency from one tissue to another [79]. In mice, NKT cells are abundant in the liver (∼30% of the T cell pool, compared with ∼1% in human) and, to a lesser extent, in the spleen and mucosal tissues. In the lung, NKT cells are resident in the blood microvasculature and extravasate to reach the parenchyma upon activation [84].

Several distinct populations of NKT cells (based on CD4, NK1.1, and IL-25 receptor expression) have been reported and differ in their tissue distribution, cytokine profile, and effector functions. Many physiological roles are fulfilled by NKT cells; they include protective immunity against pathogens, tumour surveillance, regulation of inflammatory diseases and the modulation of innate and adaptive immune responses [78], [79], [85]. These functions are mainly mediated by cytokine secretion, although NKT cells also exert cytotoxic properties. The multifunctional properties of NKT cells are primarily due to the presence of distinct NKT subsets, the cytokine environment and the nature of the Ag itself and the Ag-presenting cell.

#### Roles during respiratory infections

Natural killer T cells respond to a wide range of microbial pathogens (from viruses to helminth parasites) (for reviews, [85]–[90]). The cells activate directly in response to microbial (mostly bacterial) CD1d-restricted lipids and/or indirectly in response to inflammatory cytokines (particularly IL-12), in conjunction with (in some cases) self-lipids [91]–[95]). In this setting, PRRs (including TLRs) expressed by sentinel cells have a major functional role (Figure 1) [91], [96]–[98]. The apparent roles of NKT cells in host defence against respiratory pathogens and in infection-associated pulmonary disorders vary according to the model studied and the nature of the initial trigger. Recent evidence indicates that lung NKT cells contribute to pathogen clearance in acute viral infections (including respiratory syncytial virus and influenza A virus [IAV] infection) [99]–[103]. It is known that NKT cells cause pulmonary eosinophilia and fibrosis after respiratory syncytial virus infection [99] but limit inflammation in the context of IAV infection [100]–[103]. Natural killer T cells have a variety of functional roles during bacterial lung infections (Figure 1); whilst the cells protect against the intracellular bacteria *Chlamydia pneumoniae*, they promote susceptibility to *C. muridarum* lung infection (a phenomenon due to the recruitment/activation of different NKT cell subsets) [104], [105]. There is evidence for a role of NKT cells in murine tuberculosis infection, although their absence is not essential to control infection [106]–[110]. In contrast, exogenous activation of NKT cells with the superagonist α-galactosylceramide protects susceptible mice from tuberculosis [111], [112]. Moreover, early NKT cell activation (via IFN-γ) has a role in resistance to *M. bovis* BCG infection in mice [113], [114]. Lastly, patients with active/acute tuberculosis have fewer peripheral NKT cells than patients with latent tuberculosis; normal NKT cell counts can be restored by treating the active tuberculosis [115], [116].

## Roles of Non-conventional T Lymphocytes in Pneumococcal Infection

### Innate immunity in the early control of *S. pneumoniae*

The early control of pneumococcal colonization/infection is a dynamic process involving a wide array of host innate factors, including natural antibodies (Abs), elements of the complement pathways, nonopsonic receptors (such as the macrophage scavenger receptor MARCO and the mannose receptor), PRRs, cytokines, and many cell types (for reviews, [6], [7], [117], [118]). *S. pneumoniae* activates a plethora of PRRs in sentinel cells, including nucleotide-binding oligomerization domain (NOD)-like receptor 2, TLRs (TLR2, TLR4 and TLR9), the cytosolic DNA sensor AIM2, and the inflammasome-forming protein NOD-like receptor family, pyrin domain-containing 3 (NLRP3) (for a review, [118]). In turn, these stimulatory pathways induce neighbouring immune and non-immune cells to produce various inflammatory mediators. Epithelial surfaces constitute the first line of defence against *S. pneumoniae* [119]. Adhesion of *S. pneumoniae* to the epithelium of the nasopharynx or within the alveoli causes local release of pro-inflammatory mediators (including cytokines, chemokines, and antimicrobial peptides), which in turn initiate innate immunity. Macrophages have a crucial role in the early control of *S. pneumoniae* through phagocytic ingestion of the bacteria (a process facilitated by opsonic Abs) [6], [7], [117], [118], [120]. Through phagocytosis and the release of oxygen radicals and antimicrobial peptides, neutrophils are also essential for innate immune responses against pneumococci. Research has also highlighted the essential contribution of NK cells to the early response to pulmonary *S. pneumoniae* infection, although they are also involved in pathogenesis (through IFN-γ) in a model of pneumococcal meningitis [121], [122]. Interferon-γ and IL-17 are the crucial T helper-type cytokines involved in the early control of *S. pneumoniae* infection [120], [123]–[132]. Soon after pneumococcal colonization and/or lower respiratory tract infection, IFN-γ and IL-17 are produced by various cell types, including “innate” T cells, NK cells, neutrophils, and at later time points, conventional T lymphocytes [94], [95], [120], [124], [126]–[132]. Pneumococcal-specific CD4^+^ T lymphocytes (and particularly those producing IL-17) have an important role in protection against bacterial carriage and pulmonary infection [120], [129], [133]–[136]. Our recent data also suggest that IL-22 (a member of the Th17-type cytokine family) might exert an early anti-pneumococcal effect [137]. Type 3 innate lymphoid cells (ILC3) constitute an innate cell population that reacts to inflammatory cytokines (e.g., IL-1β/IL-23) and is thought to be strongly involved in antimicrobial defence and tissue repair in intestines (for a review, [138]). It is noteworthy that pulmonary ILC3 produce IL-22 during *S. pneumoniae* infection [137].

There is a growing body of evidence for an early-onset role of “innate” T cells in pneumococcal immunity. Elucidation of the roles of MAIT cells, γδ T cells, and NKT cells (all of which produce IL-17 and IFN-γ) in pneumococcal infection may not only provide an in-depth understanding of host defence mechanisms and disease pathogenesis but also accelerate the development of novel efficient therapies.

### Potential roles of MAIT cells in pneumococcal infection

The activation status and functions of MAIT cells during pneumococcal infection have not yet been characterized. Three MR1-binding metabolites of the riboflavin pathway activate MAIT cells, namely, reduced 6-hydroxymethyl-8-d-ribityllumazine, 7-hydroxy-6-methyl-8-d-ribityllumazine, and to a lesser extent, the latter's precursor, 6,7-dimethyl-8-d-ribityllumazine [27]. It is not known whether *S. pneumoniae* activates MAIT cells via MR1. However, the genomic analysis of various *S. pneumoniae* serotypes and strains indicates that these bacteria might express enzymes involved in the synthesis of riboflavin metabolites [139], [140]. In contrast, and in line with the observation that group A streptococci fail to activate MAIT cells [28], *S. pyogenes* lacks 3,4-dihydroxy-2-butanone 4-phosphate synthase and 6,7-dimethyl-8-ribityllumazine synthase [141], [142], two critical enzymes involved in the conversion of ribulose 5-phosphate to MAIT cell-activating Ags. This suggests that *S. pneumoniae* may produce MR1 ligands able to activate MAIT cells and that the latter might play a part in the early recognition of pneumococci. Through their capacity to produce IFN-γ, as well as IL-17 [24], [143], MAIT cells might contain pneumococcal infection. This hypothesis must be tested in future studies.

### Roles of γδ T cells in pneumococcal infection

The potential role of γδ T cells in pneumococcal infection has only been studied in animal models. Most experiments have used the frequently colonizing, invasive serotype 3 (Figure 1). In the mouse system, γδ (Vγ1^+^, Vγ4^+^, Vγ6^+^) T cells accumulate and activate in the lungs during *S. pneumoniae* infection [144], [145]. It is noteworthy that mice lacking γδ T cells display a higher bacterial load in the lung and have a lower survival rate than wild-type controls [130], [132], [144]. Gamma delta T cell deficiency is associated with defective secretion of MIP-2, TNF-α, and IL-17 and poor recruitment of neutrophils [130], [132], [144]. It is likely that γδ T cells contribute to neutrophilia and antipneumococcal defenses through IL-17 production [126], [130], [132]. To a lower extent [126], [132], γδ T cells also produce IFN-γ in the context of infection by *S. pneumoniae* serotype 3 and serotype 1 (an infrequently colonizing but invasive serotype), although the significance of this observation has yet to be established ([145] and our unpublished data). The mechanisms responsible for γδ T cell activation are still elusive and might rely on the production of self, stressed-induced, or pneumococcal ligand(s) and/or the local synthesis of inflammatory cytokines including IL-12 plus IL-18 (IFN-γ) and IL-1β plus IL-23 (IL-17) (Figure 1). Pneumolysin may synergize with pneumococcal and endogenous danger signals to trigger the secretion of inflammatory cytokines through TLR and NLRP3 activation [126]. Generation of Abs to pneumococcal capsular polysaccharides is important in the control of *S. pneumoniae* (i.e. opsonization and complement fixation). To date, no study has yet investigated the natural role of γδ T cells in Ab formation during the course of *S. pneumoniae* infection. Along with their role in early defence against *S. pneumoniae*, γδ T cells participate in the resolution phase of pneumococcal pneumonia by eliminating inflammatory mononuclear phagocytes [146].

Respiratory viral infections can lead to secondary pneumococcal infections following alterations in the tract's mechanical and immunological defences [10], [11], [147], [148]. Since γδ T cells are critical for host defense against *S. pneumoniae* [130], [132], [144], it was suggested that respiratory virus infections may inhibit γδ T cell activation and functions in the lung. Of interest, γδ T cells from influenza-experienced mice are less able to produce IL-17 upon challenge with *S. pneumoniae*—a phenomenon that depends on the IFN type I/IL-27 axis and leads to enhanced pneumococcal susceptibility [131], [132]. It is tempting to speculate that targeting γδ T cells cells to boost immunity against pneumococcal infection might help to limit post-influenza bacterial infection. Unfortunately, ligands capable of specifically activating γδ T cells have not yet been described in the mouse system. It is therefore not possible to study the effects of exogenous γδ T cell activation on host defence in the context of murine pneumococcal infection.

### Roles of NKT cells in pneumococcal infection

Several studies have highlighted the role of lung NKT cells in host defence against *S. pneumoniae* (Figure 1). When infected with *S. pneumoniae* (serotype 3), NKT-cell-deficient mice exhibit a higher mortality rate and bacterial load in the lung than wild-type controls [149]. In the absence of NKT cells, the early recruitment of neutrophils is impaired by a lack of MIP-2 secretion. It has been suggested that NKT-cell–derived IFN-γ has a critical role in protection against pneumococcal pneumonia [125]. Exogenous administration of recombinant IFN-γ restored the ability of NKT-cell-deficient mice to eliminate bacteria from the lung. It is noteworthy that the administration of IFN-γ led to the production of MIP-2 and TNF-α, which in turn promoted neutrophil recruitment to the infected area. Two other studies have confirmed the natural protective role of NKT cells in animal models of *S. pneumoniae* serotype 3 infection, although the underlying mechanisms were not studied. Using *S. pneumoniae* serotype 1, we have also found that NKT cells are important innate effectors in the early clearance of pneumococci (our unpublished data).

**Figure 2:**
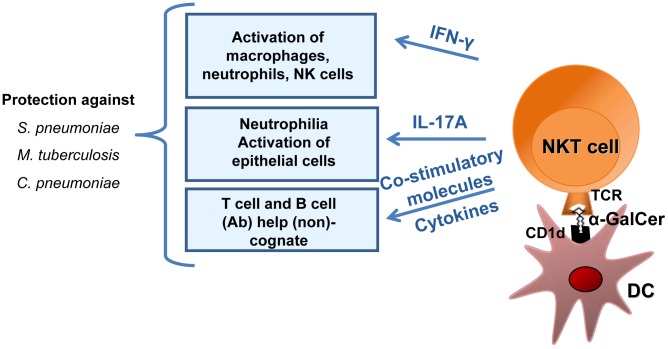
Mechanisms of NKT cell–based antibacterial immunity in response to exogenous α-galactosylceramide activation. α-galactosylceramide (α-GalCer) presented by respiratory DCs in the context of CD1d activates pulmonary NKT cells to produce IFN-γ and IL-17, which in turn activate macrophages, neutrophils, and possibly NK cells and epithelial cells. Since NKT cells can provide help to conventional T lymphocytes and B lymphocytes, it is likely that NKT cell activation by α-galactosylceramide not only controls the bacterial burden early after infection but also promotes memory protective immune responses against secondary respiratory bacterial infections.

Mouse NKT cells produce IFN-γ and (to a much lower extent) IL-17 in the natural course of *S. pneumoniae* (serotypes 1 and 3) infection ([94], [95], [126], [132], our unpublished data). The mechanism leading to NKT cell activation is now well understood (Figure 2). In *S. pneumoniae* serotype 3, the cell wall contains α-glucosyldiacylglycerol—a glycolipid that is structurally similar to the canonical NKT cell activator α-galactosylceramide. It is noteworthy that this glycolipid activates NKT cells in a CD1d-restricted manner [94]. Moreover, *S. pneumoniae* activates the release of IL-12 by DCs, a phenomenon contributing to IFN-γ production by NKT cells [95], [150]. In contrast to the situation with γδ T cells, it is possible to study the effects of exogenous NKT cell activators on host responses in mice. Prophylactic administration of α-galactosylceramide protects against lethal *S. pneumoniae* serotype 1 and 3 infections [125], [149], [151]. Our findings suggest a critical role for IFN-γ, IL-17, and neutrophils (but not alveolar macrophages) in pneumococcal clearance (Figure 2) [151]. No studies have yet addressed the potential role of NKT cells in human pneumococcal infections.

**Figure 3:**
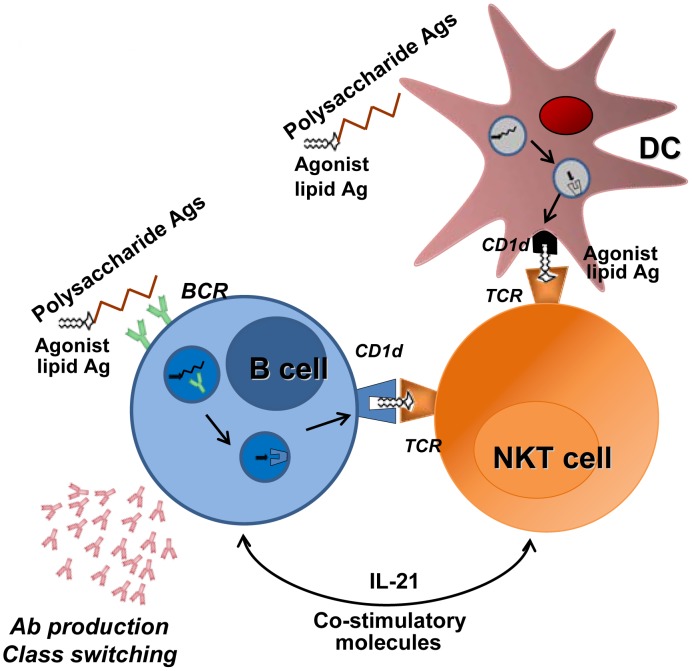
Promotion of B cell responses by cognate NKT cells help. B cells recognize, through their B cell receptor (BCR), capsular polysaccharide Ags associated with lipids (e.g., formulated particles). BCR crosslinking leads to endocytosis and transport of Ags to endosomal compartment. After endosomal/lysosomal digestion, lipid Ags bind to the CD1d molecule. The cell surface CD1d-lipid complex activates NKT cells through TCR engagement, which in turn provide help to B cells. In this setting, NKT cells must be primed by DCs before interacting with B cells. NKT cells favor B cell responses (Ab production and class switching) through IL-21 secretion and co-stimulatory molecules. The ability of NKT cells to promote cognate-dependent B cell responses might be instrumental in the formulation of new vaccines against T cell–independent Ags such as pneumococcal capsular polysaccharides. Of note, during infection, natural pneumococcal NKT cell ligands might also promote Ab responses through cognate NKT cell help to B cells.

Natural killer T cells can indirectly or directly help B cells to mount Ab responses [152]–[157]. Although this activity has not yet been studied in the context of pneumococcal infection, NKT cells might indirectly help B cells through DC licensing and enhanced priming of conventional CD4 T cells (as this is the case for indirect help in other infectious systems) [78], [85]. In parallel, the synthesis of NKT cell agonist by *S. pneumoniae* suggests the existence of direct help by NKT cells. It is noteworthy that NKT cells have a crucial function in the production of antipneumococcal Abs and class switching in response to pneumococcal polysaccharide vaccines [158], [159]. In humans, there is a positive correlation between the peripheral NKT cell count and the production of IgG after administration of the 23-valent polysaccharide vaccine [160]. Remarkably, the use of liposomal nanoparticles displaying synthetic NKT-cell-activating lipids and polysaccharides (mimicking natural pneumococcal Ags) results in the generation of long-lasting memory B cells and efficient isotype switch [161]. The mechanism leading to Ab production and class switching requires the recognition of lipid and capsular polysaccharide Ags by NKT cells and B cells, respectively, and involves cognate NKT-B cell conjugate formation (direct help, Figure 3). In the future, it might be possible to exploit this unique property (i.e., direct help) and thus optimize Ab responses against T cell-independent pneumococcal polysaccharide Ags [161], [162].

## Conclusions and Future Directions

There is a now a consensus on the importance of γδ T cells and NKT cells in animal models of pneumococcal infection. However, no study has yet addressed the potential role of MAIT cells during pneumococcal infection, and so further research is required. *Streptococcus pneumoniae* might have a functional riboflavin-biosynthesis pathway and thus the potential to activate MAIT cells. Now that MAIT Ags have been identified, the use of MR1-Ag tetramers will enable the characterization and tracking of MAIT cells during pneumococcal infections in animal models and in humans. Since a growing body of evidence suggests that MAIT cells are involved in the early recognition and containment of microbial infection, these Ag-experienced effector T cells are likely to exert important functions during pneumococcal colonization and/or infection. If so, manipulation of these cells might be of value. Although the role of MAIT cells in acquired responses has yet to be established, it might also be worth looking at whether these cells could be candidates for vaccine targeting.

In the mouse system, γδ T cells and NKT cells act as key elements in antipneumococcal immunity through the production of Th1 and/or Th17-related cytokines. However, several important questions must be answered before these cells can be exploited as potential targets for anti-pneumococcus immunotherapies. For instance, a more comprehensive understanding of their precise modes of activation and their functions is essential. Moreover, the γδ T cells' and NKT cells' respective roles in pneumococcal pneumonia are still elusive. By controlling tissue damage and/or promoting tissue repair processes (via IL-22 and growth factors), these cells may be important in the control of lung pathogenesis. Along the same lines, the potential role(s) of γδ T cells and NKT cells in pneumococcal colonization versus lung and/or systemic invasion merits further investigation. In view of their diverse and sometimes opposing functions, the respective roles of γδ T cell and NKT cell subsets have yet to be characterized. In this context, the development of relevant animal models (enabling specific depletion of γδ cell and NKT cell subsets) and the discovery of novel ligands would be important breakthroughs in this field. In parallel, the use of humanized mice and nonhuman primates is likely to (i) provide highly useful information on the role of these non-conventional T cells during pneumococcal infection and (ii) speed the design of preventive or curative γδ T cell– and NKT cell–based immunotherapies.

There are currently no data on the roles of γδ T cells and NKT cells in pneumococcal infections in humans. Large-scale clinical and genetic studies are clearly warranted, in order to (for instance) link γδ T cell and NKT cell (dys)functions to the human lung diseases associated with pneumococcal infection. Could γδ T cells and/or NKT cells be attractive prophylactic/therapeutic targets for preventing and/or treating pneumococcal infections? This question is an important one, in view of the many situations in which the (innate) immune system is compromised as a consequence of cancer, trauma, immunosuppressive drug treatment, sepsis, chronic inflammation or prior infections. Preclinical data suggest that γδ T cell functions are impaired in the context of influenza—an effect that could (at least in part) account for secondary pneumococcal infection. In this context, targeting γδ T cells via phosphoAgs (to assist immunity against pneumococcal infection) might be a useful approach (in combination with antibiotic treatment) for limiting pneumonia- and/or bacteraemia-associated mortality in patients. The same strategy might also be of value in the case of NKT cells and MAIT cells. Furthermore, the adjuvant properties of γδ T cells and/or NKT cells might be exploited in the design of more efficient antipneumococcal vaccines. Ligands for these cells present many advantages over conventional adjuvants and might conceivably be used to optimize the magnitude and duration of the adaptive immune response. Indeed, γδ T cells and NKT cells can directly activate DCs through non-PRR mechanisms; this unique interplay might not only fine-tune immune responses but also extend the magnitude and duration of the memory T and B cell responses. Lastly, optimized particulate vaccines containing conjugated pneumococcal polysaccharides and non-conventional T cell agonists might perform better than today's poorly immunogenic B cell carbohydrate vaccines.
